# Segmental redistribution of myocardial blood flow after coronary sinus reducer implantation demonstrated by quantitative perfusion cardiovascular magnetic resonance

**DOI:** 10.1016/j.jocmr.2025.101868

**Published:** 2025-02-26

**Authors:** Kevin Cheng, Francisco Alpendurada, Chiara Bucciarelli-Ducci, Jose Almeida, Peter Kellman, Jonathan M. Hill, Dudley J. Pennell, Ranil de Silva

**Affiliations:** a National Heart and Lung Institute, Imperial College London, London, UK; b Royal Brompton and Harefield Hospitals, Guy’s and St Thomas’ NHS Foundation Trust, London, UK; c School of Biomedical Engineering and Imaging Sciences, Faculty of Life Sciences and Medicine, King’s College University, London, UK; d Division of Intramural Research, National Heart Lung and Blood Institute, Bethesda, Maryland, USA

**Keywords:** Refractory angina, Angina pectoris, Coronary sinus reducer, Cardiovascular magnetic resonance imaging, Quantitative perfusion, Myocardial blood flow

## Abstract

**Background:**

The coronary sinus reducer (CSR) is a novel percutaneous treatment for patients with refractory angina. Increasing evidence supports its clinical efficacy in patients with advanced epicardial coronary artery disease. However, its mechanism of action and its effects on myocardial perfusion remain undefined. Using quantitative stress perfusion cardiovascular magnetic resonance (CMR), this study assessed changes in myocardial perfusion in patients with refractory angina undergoing CSR implantation.

**Methods:**

This single-center retrospective observational cohort study included 16 patients. Rest and adenosine stress perfusion CMR was performed before and at median 5 months after CSR implantation. Perfusion images were acquired using a dual-sequence quantitative protocol with automated generation of myocardial blood flow (MBF; mL/min/g). In addition to visual assessment of ischemic segments, changes in absolute MBF across myocardial segments and between myocardial layers were analyzed.

**Results:**

A high proportion of myocardial segments had visually adjudicated ischemia at baseline (208 out of 254: 81.9%), which significantly reduced after CSR implantation (175 out of 254: 68.9%; P = 0.001). There were no changes in global MBF or strain values. Changes in myocardial perfusion reserve (MPR) correlated with baseline MPR with more ischemic segments at baseline improving to a greater extent at follow-up. Similar patterns were observed in both the left and right coronary artery territories. Changes in endocardial/epicardial MBF ratio at stress were similarly dependent on baseline values.

**Conclusion:**

In patients with refractory angina undergoing CSR implantation, quantitative stress perfusion CMR demonstrated redistribution of myocardial perfusion across segments, from less ischemic to more ischemic myocardium, and across myocardial layers with greatest improvements in endocardial perfusion observed in the most ischemic myocardium. Further studies are needed to validate the different patterns of MBF redistribution that may occur after CSR implantation and correlate with clinical outcomes.

## Graphical abstract

**Figure:**
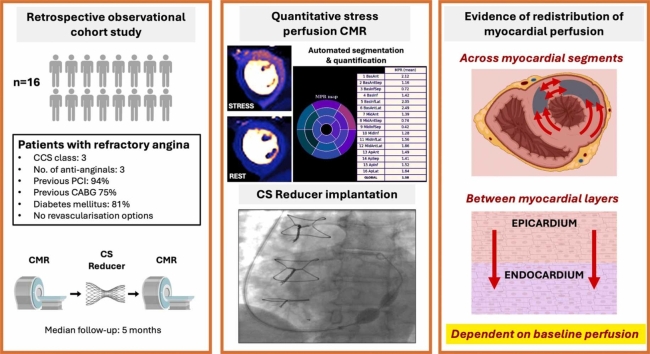

## 1 Introduction

The coronary sinus reducer (CSR) is a novel device that improves symptoms and quality of life in patients with refractory angina secondary to advanced coronary artery disease (CAD) [1], [2], [3], [4], [5]. The COSIRA trial, the first randomized, double-blinded, sham-controlled trial of CSR implantation, demonstrated improvement in angina by Canadian Cardiovascular Society (CCS) score and quality of life by Seattle Angina Questionnaire, but failed to demonstrate improvements in ischemic burden by stress echocardiography [1]. The ORBITA-COSMIC study confirmed significant angina reduction after CSR implantation compared with sham, using app-based patient reporting of angina, but failed to meet its primary efficacy outcome of improvement in stress myocardial blood flow (MBF) in visually adjudicated ischemic segments by quantitative stress perfusion cardiovascular magnetic resonance (CMR) [2].

Despite increasing evidence of the clinical benefit of this therapy for patients with refractory angina, the underlying mechanisms remain under investigation. Several studies using CMR have shown changes in myocardial perfusion reserve index (MPRI), a semi-quantitative metric [6], [7], [8], and more recently endocardial to epicardial (endo/epi) stress MBF ratio using fully quantitative stress perfusion CMR [2]. Redistribution of MBF toward less well-perfused segments at baseline has also been demonstrated after CSR implantation by Rubidium-82 positron emission tomography [9], [10]. We tested the hypothesis that CSR implantation redistributes MBF using state-of-the-art fully automated pixel-wise in-line quantitative myocardial perfusion mapping CMR.

## 2 Methods

In a single-center retrospective observational study of patients with refractory angina due to obstructive epicardial CAD and no further revascularization options, rest and adenosine (140-210 mcg/kg/min) stress perfusion CMR was performed before and at median 5 months [IQR: 4-6.75] after CSR implantation. Patients were asked to abstain from caffeine for at least 24 h before the scans, which were performed on 1.5T scanners (Aera/Sola/Avanto Fit, Siemens Healthineers, Erlangen, Germany) using a standardized protocol that included cine imaging, adenosine stress and rest perfusion, and late gadolinium enhancement (LGE) imaging. Intravenous adenosine was infused according to a standard clinical protocol to achieve maximal hyperemia [11]. Basal, mid-ventricular, and apical short-axis perfusion images were acquired using a dual-sequence protocol described previously [12]. Quantification of rest and stress MBF (mL/min/g) and myocardial perfusion reserve (MPR, calculated as MBF_STRESS_/MBF_REST_) values used an artificial intelligence approach that automatically segmented the myocardium into the 16-segment American Heart Association model (examples provided in Supplementary Fig. 1) [13]. Contouring with this approach is fully automated without need for user input leading to implicit blinding of perfusion data to other CMR and demographic parameters. MBF values were further categorized by myocardial layer (endocardial or epicardial). The reproducibility of this quantitative approach has been previously shown [14]. LGE was performed at least 5 min after the second gadolinium bolus injection for stress perfusion imaging, using phase-sensitive inversion recovery sequences [15].

All scans were analyzed using CVI42 (Circle Cardiovascular Imaging, Calgary, Alberta, Canada), and visual adjudication of ischemic segments and scar burden was performed by an experienced CMR level 3 certified reporter according to established reporting guidelines [16]. MBF_REST_ values were corrected by the average heart rate. Endo/epi ratio was calculated as the ratio of endocardial to epicardial MBF. Perfusion changes were calculated as the pairwise difference between baseline and follow-up values in each segment.

Fisher’s exact test was used to compare proportions and Wilcoxon matched-pairs sign rank tests to compare paired patient-level global values. Linear mixed-effects models with random slopes and intercepts were used for segmental analyses. This study was independently approved by the United Kingdom Health Research Authority (23/HRA/1136).

## 3 Results

Sixteen patients were included and baseline patient demographics are shown in Table 1. Previous rates of revascularization were high (percutaneous coronary intervention [PCI]: 94% (15 out of 16); coronary artery bypass grafting [CABG]: 75% (12 out of 16) and 81% (13 out of 16) of patients had diabetes mellitus. All CSR implants were performed without complication. Haemodynamic data during perfusion CMR acquisition are presented in Supplementary Table 1.

**Table 1:** Baseline demographics of the study population.

Median age			65 [58.3-70.8]
Sex		Male	14 (88%)
		Female	2 (12%)
Hypertension			13 (81%)
Diabetes mellitus			13 (81%)
Hyperlipidaemia			14 (88%)
Previous CABG			12 (75%)
Previous PCI			15 (94%)
Smoking (current/ex)			9 (56%)
Median left ventricular systolic function			63.8% [54.9-69.8]

Median CCS class			3 [3-3]
CCS 3			14 (88%)
CCS 4			2 (12%)

Median number of anti-anginal medications			3 [2.75-4]
Baseline number of anti-anginal medication	≤1		0 (0%)
	2		4 (25%)
	3		5 (31%)
	≥4		7 (44%)
*Anti-anginal medications: beta-blockers, calcium channel blockers, long-acting oral nitrates, ranolazine, nicorandil, trimetazidine, ivabradine*			

Baseline disease-modifying therapy	Aspirin		15 (94%)
	P2Y_12_ antagonist		12 (75%)
	Statin		16 (100%)
	ACEi/ARB		13 (81%)

*ACEi* angiotensin-converting enzyme inhibitor, *ARB* angiotensin receptor blocker, *CABG* coronary artery bypass grafting, *CCS* Canadian Cardiovascular Society, *PCI* percutaneous coronary intervention. Values are numbers (%) of cases or medians (interquartile range).

Median CCS class was 3 [3-3] at baseline and 2 [1-3] at follow-up (median difference: −1; P = 0.007). No patient had altered anti-anginal medication or additional revascularization during follow-up. Baseline total scar burden was low (7.4% [3.4-10.8%]), and from baseline to follow-up, there was no change in left ventricular ejection fraction (63.8% [54.9-69.8] vs 65.2% [57.3-70.2], P = 0.25) or three-dimensional global strain (radial: 30.4% [26.4-33.5] vs 32.6% [28.2-38.2], P = 0.12; circumferential: −17.4% [−19.4 to −14.7] vs −18.0% [−18.6 to −14.8], P = 0.56; longitudinal: −11.7% [−13.5 to −9.19] vs −11.7% [−12.8 to −7.77], P = 0.43).

**Fig. 1:**
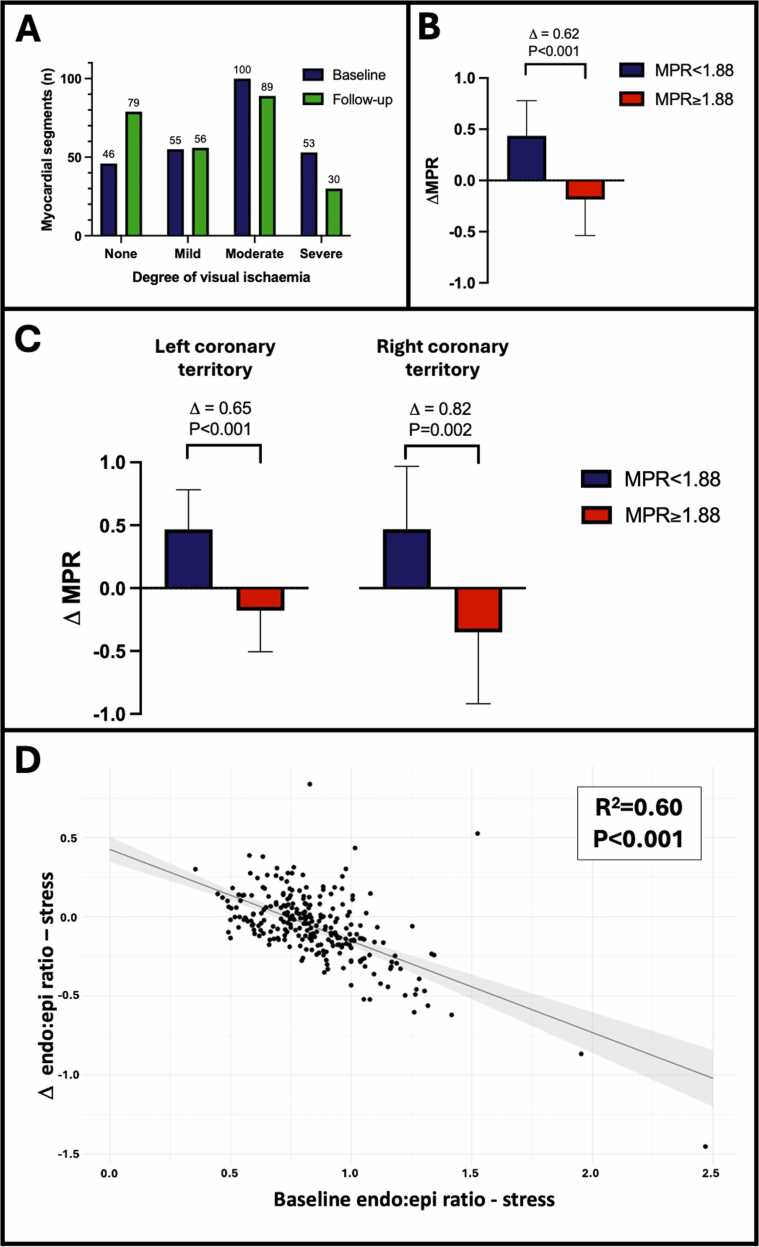
(A) Graph showing the number of myocardial segments and the degree of visually adjudicated ischemia at baseline and after CSR implantation. (B) Stratified analysis showing differential effects in ΔMPR dependent on baseline MPR. (C) Similar changes in ΔMPR dependent on baseline MPR were observed in both the left and the right coronary artery territories. (D) The magnitude of change in stress endo/epi ratio correlated with baseline values. *CSR* coronary sinus reducer, *MPR* myocardial perfusion reserve

Quantitative analysis could be performed in 254 out of 256 segments. Partial thickness scar was present in 41 segments and full thickness scar in 9 segments. Visual ischemia at baseline was present in the majority of segments (208 out of 254: 81.9%). Severity of ischemia included none: 46 segments (median MPR = 2.42 [2.02-3.04]); mild: 55 segments (MPR = 1.88 [1.52-2.45]); moderate: 100 segments (MPR = 1.60 [1.19-2.19]); severe: 53 segments (MPR = 0.96 [0.80-1.31]). Compared to baseline, there were fewer visually ischemic segments at follow-up (175 out of 254: 68.9%) with significant improvement in the severity of visual ischemia (P = 0.001; Fig. 1A).

Quantitative analysis demonstrated no change in global MPR (1.67 [1.33-2.38] vs 1.70 [1.41-2.55]; P = 0.40), global stress MBF (1.43 [0.91-1.70] vs 1.29 [1.17-1.50] mL/min/g; P = 0.78) or global rest MBF (0.74 [0.59-1.02] vs 0.73 [0.64-0.89] mL/min/g; P = 0.87) from baseline to follow-up (Supplementary Fig. 2). When dividing segments into two groups, above and below a threshold of MPR <1.88 (based on the median MPR in segments with mild ischemia visually adjudicated at baseline), differential effects on change (Δ) in MPR were observed. In segments with baseline MPR <1.88, ΔMPR increased +0.44 whereas in segments with baseline MPR ≥1.88, ΔMPR decreased −0.19 (P < 0.001; Fig. 1B). Similar differential effects in ΔMPR were observed in both the left (baseline MPR <1.88: ΔMPR increased +0.47; MPR ≥1.88: ΔMPR decreased −0.18; P < 0.001) and right (baseline MPR <1.88: ΔMPR increased +0.47; MPR ≥1.88: ΔMPR decreased −0.35; P < 0.002) coronary territories (Fig. 1C).

In a segmental analysis, baseline MPR significantly correlated with ΔMPR (R^2^ = 0.79; P = 0.001) such that in segments with a lower baseline MPR, a greater increase in ΔMPR occurred after CSR implantation. Conversely, in segments with higher baseline MPR, ΔMPR at follow-up decreased. A similar effect was observed for redistribution of perfusion by myocardial layer, such that the degree of change in endo/epi ratio at stress (RATIO_STRESS_) was associated with baseline RATIO_STRESS_. Segments with lower baseline RATIO_STRESS_ experienced greater increases in ΔRATIO_STRESS_ after CSR implantation and vice versa (R^2^ = 0.60; P < 0.001; Fig. 1D). This effect was also observed in an analysis only of visually ischemic segments (Supplementary Fig. 3).

## 4 Discussion

In this observational pilot study, redistribution of myocardial perfusion was observed from better to less well-perfused myocardial segments, an effect which was differentially observed across transmural myocardial layers. Changes in RATIO_STRESS_ were dependent on baseline RATIO_STRESS_ suggesting greater redistribution toward the endocardium in the most ischemic myocardium as an effect of CSR implantation in patients with refractory angina secondary to advanced CAD. Similar observations were present in both left and right coronary artery territories. These findings are consistent with those previously reported using CMR quantitative myocardial perfusion mapping [12] and visual assessment [6], [8].

We did not observe changes in global MPR, global stress, or global rest MBF after CSR implantation, similar to previous studies [2], [9], but the segmental analysis showed increases in the quantitative perfusion metrics in those segments most hypoperfused at baseline. These data collectively support redistribution of myocardial perfusion toward ischemic myocardium as a mechanism underlying the symptomatic improvements observed in the trials and clinical registries of CSR implantation in patients with refractory angina [1], [2], [3], [4], [5]. Interestingly, the global MPR threshold (1.88) used in the current analysis was similar to that previously reported in a study that used the same CMR methodology to measure global MBF in coronary territories supplied by a coronary artery that had evidence of physiologically significant epicardial CAD by pressure-wire assessment [17].

In this study, a potential explanation for the above relationships could be regression to the mean. However, recent data from the ORBITA-COSMIC trial, which compared CSR against a sham control, demonstrated that RATIO_STRESS_ improved in visually ischemic segments after CSR implantation. In addition, redistribution of myocardial perfusion as an effect of CSR implantation is supported by computational modeling studies [18]. Our data further suggest that the magnitude of redistribution toward the endocardium is greatest in those segments with the most reduced RATIO_STRESS_ at baseline. Other studies have suggested differing effects on redistribution of perfusion within myocardial layers when assessed by semi-quantitative MPRI after CSR implantation. Giannini et al. observed greater improvements in MPRI in the epicardium compared to the endocardium [6] while Palmisano et al. showed preferential improvements in the endocardium in ischemic segments [8].

The mechanisms by which CSR implantation leads to redistribution of myocardial perfusion remain under investigation. Recent studies using invasive coronary physiology assessment of microvascular function have suggested reductions in microvascular resistance after CSR implantation and elevation of coronary sinus pressure, which may underlie the observed improvements in myocardial perfusion and reduction in ischemic burden [19], [20], [21], [22]. The mechanisms responsible for reduced microvascular resistance remain to be determined, but have been hypothesized to include collateral recruitment, increasing capillary recruitment and transit time, improved coronary cardiac coupling, neovascularization, or their combination [23].

## 5 Conclusion

We provide novel hypothesis-generating data on the mechanism of CSR implantation and show redistribution of myocardial perfusion toward the most ischemic segments, particularly in the endocardium, as the basis for relief of ischemia and reduction of anginal symptoms. This exploratory study is limited by its open-label unblinded design, small sample size, and observational nature. Further confirmatory studies are needed and additional clarification will be provided by the results of an ongoing randomized double-blinded sham-controlled study of CSR (REMEDY-PILOT [NCT05492110], which integrates quantitative non-invasive perfusion assessment by CMR with invasive epicardial and microvascular physiology assessment.

## Funding

10.13039/501100000274British Heart Foundation Clinical Research and Training Fellowship (FS/CRTF/22/24369) and Clinical Study Grant (CS/F/20/35120).

## Author contributions

**Francisco Alpendurada:** Writing – review & editing, Visualization, Supervision, Investigation. **Kevin Cheng:** Writing – review & editing, Writing – original draft, Visualization, Software, Project administration, Methodology, Investigation, Formal analysis, Data curation, Conceptualization. **Jose Almeida:** Writing – review & editing, Investigation. **Chiara Bucciarelli-Ducci:** Writing – review & editing, Supervision. **Jonathan M. Hill:** Writing – review & editing, Supervision, Investigation. **Peter Kellman:** Writing – review & editing, Resources. **Ranil de Silva:** Writing – original draft, Validation, Supervision, Resources, Project administration, Methodology, Investigation, Formal analysis, Conceptualization. **Dudley J. Pennell:** Writing – review & editing, Supervision, Resources, Formal analysis.

## Declaration of competing interests

The authors declare the following financial interests/personal relationships which may be considered as potential competing interests: Ranil de Silva reports a relationship with Shockwave Medical Inc. that includes funding grants and speaking and lecture fees. Ranil de Silva reports a relationship with Abbott Vascular Inc. that includes funding grants and speaking and lecture fees. Dudley J. Pennell reports a relationship with Siemens Healthineers AG that includes funding grants. Chiara Bucciarelli-Ducci reports a relationship with Society of Cardiac Magnetic Resonance that includes employment. Chiara Bucciarelli-Ducci reports a relationship with Circle Cardiovascular Imaging Inc. that includes speaking and lecture fees. Chiara Bucciarelli-Ducci reports a relationship with Siemens Healthineers AG that includes speaking and lecture fees. Chiara Bucciarelli-Ducci reports a relationship with Bayer AG that includes speaking and lecture fees. Chiara Bucciarelli-Ducci reports a relationship with Philips that includes speaking and lecture fees. Chiara Bucciarelli-Ducci reports a relationship with GE Healthcare that includes speaking and lecture fees. Jonathan Hill reports a relationship with Abbott that includes funding grants and speaking and lecture fees. Jonathan Hill reports a relationship with AbioMed Inc. that includes funding grants and speaking and lecture fees. Jonathan Hill reports a relationship with Boston Scientific Corporation that includes funding grants and speaking and lecture fees. Jonathan Hill reports a relationship with Shockwave Medical Inc. that includes funding grants and speaking and lecture fees. Jonathan Hill reports a relationship with Shockwave Medical Inc. that includes equity or stocks. The other authors declare that they have no known competing financial interests or personal relationships that could have appeared to influence the work reported in this paper.
