# Supplementary material for: Segmental redistribution of myocardial blood flow after coronary sinus reducer implantation demonstrated by quantitative perfusion cardiovascular magnetic resonance
Source: J Cardiovasc Magn Reson. 2025 Feb 26;27(1):101868. doi: 10.1016/j.jocmr.2025.101868 (PMC12033896; doi:10.1016/j.jocmr.2025.101868)
Supplement: Supplementary file 1 — Supplementary material [file mmc1.docx]

**Supplementary Figure 1.** Example CMR perfusion images at baseline (A, B, C) and follow-up (D, E, F) after CSR implantation. **A & D)** Quantitative myocardial perfusion colour maps. **B & E)** Automated in-line segmentation. **C & F)** Quantification of stress MBF, rest MBF and MPR (left to right).


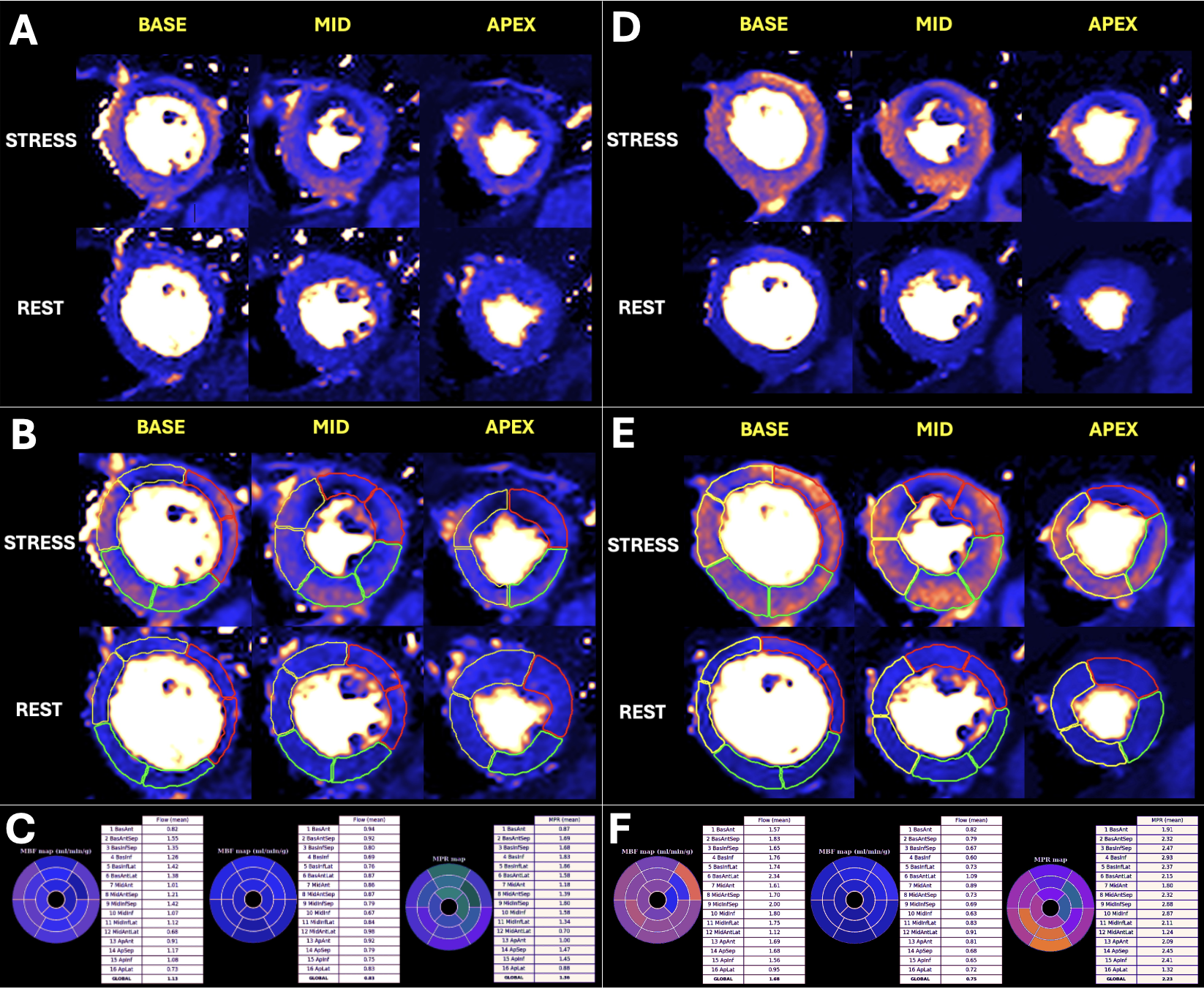


**Supplementary Figure 2.** Patient-level changes in quantitative stress perfusion CMR metrics before and after CSR implantation. **A)** Myocardial perfusion reserve (MPR). **B)** Stress myocardial blood flow (MBF). **C)** Rest myocardial blood flow. **D)** Endocardial:epicardial ratio at stress.

**P=0.40**

**P=0.78**

**P=0.87**

**P=0.21**

**Supplementary Figure 3. Linear mixed effects model showing the relationship between change in endocardial:epicardial ratio at stress and its baseline value, only in visually-adjudicated ischaemic segments.**

**
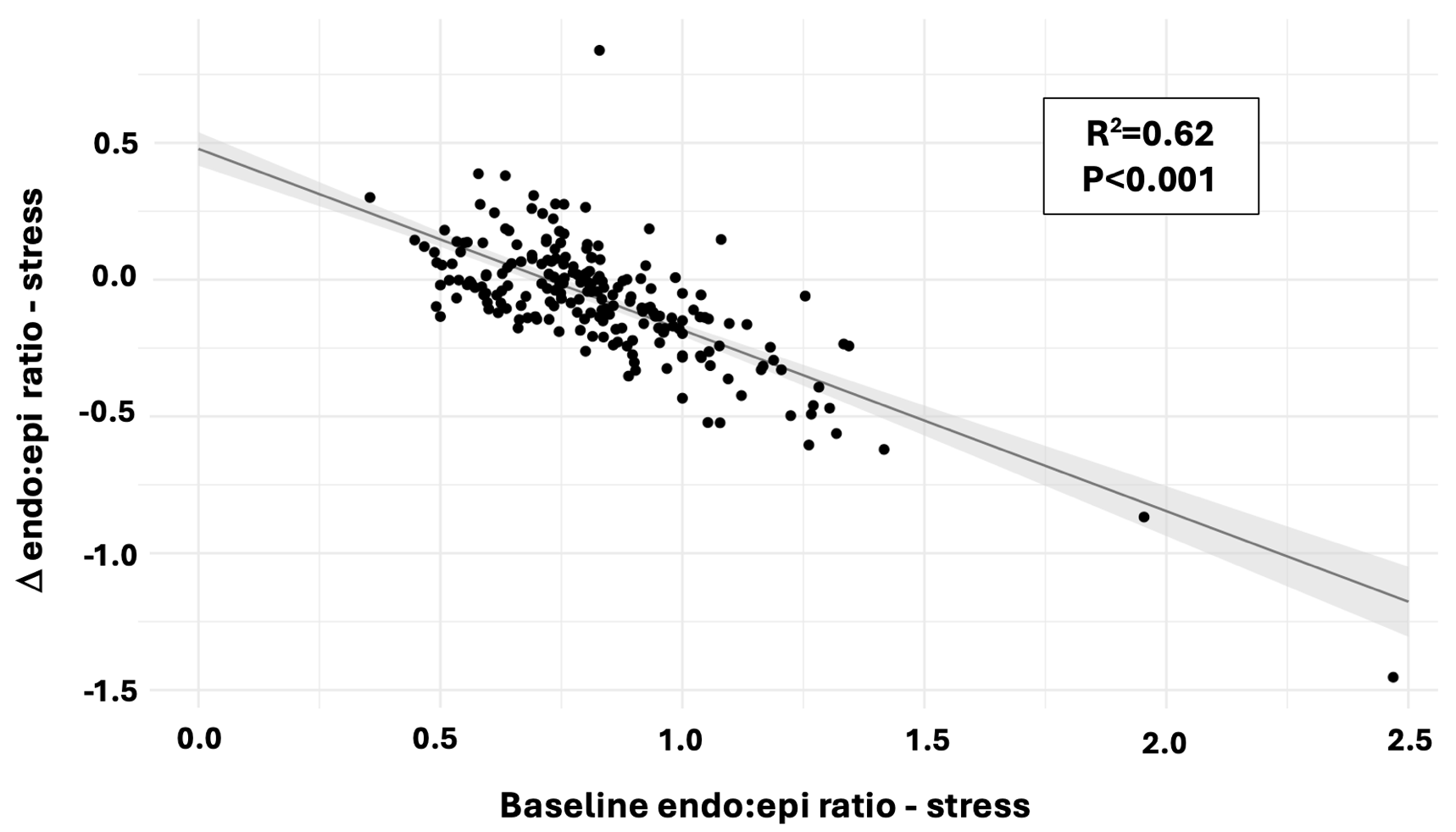
**

**Supplementary Table 1.** Haemodynamic data during perfusion CMR acquisition.

|  | **All** | **Baseline** | **Follow-up** | **P-value (BL:FU)** |
| --- | --- | --- | --- | --- |
| **Rest HR** (bpm) | 62 (58-66) | 61 (57-68) | 63 (58-66) | 0.58 |
| **Rest SBP** (mmHg) | 134 (123-151) | 139 (113-151) | 132 (124-149) | 0.41 |
| **Rest RPP**  (mmHg x bpm) | 8246  (7153-10105) | 8247  (7153-10387) | 8246  (7103-8931) | 0.74 |
| **Stress HR** (bpm) | 83 (75-87) | 82 (70-91) | 84 (76-87) | 0.94 |
| **Stress SBP** (mmHg) | 124 (113-139) | 128 (106-143) | 119 (113-127) | 0.37 |
| **Stress RPP**  (mmHg x bpm) | 9844  (8584-11480) | 10294  (8584-11790) | 9831  (8069-10591) | 0.27 |
| **Change HR** (bpm) | +18 (11-25) | +20 (15-26) | +18 (10-24) | 0.67 |
| **Change SBP** (mmHg) | -9 (-23-0) | -8 (-23-0) | -12 (-24-0) | 0.90 |
| **Change RPP**  (mmHg x bpm) | +1383  (140-2989) | +2162  (437-2989) | +1236  (-263-2784) | 0.56 |
| BL: baseline; FU: follow-up; HR: heart rate; RPP: rate-pressure product; SBP: systolic blood pressure | | | | |
